# Distinct Cerebral Pathways for Object Identity and Number in Human Infants 

**DOI:** 10.1371/journal.pbio.0060011

**Published:** 2008-02-05

**Authors:** Véronique Izard, Ghislaine Dehaene-Lambertz, Stanislas Dehaene

**Affiliations:** 1 INSERM, U562, Cognitive Neuroimaging Unit, F-91191 Gif/Yvette, France; 2 CEA, DSV/I^2^BM, NeuroSpin Center, Gif/Yvette, France; 3 Université Paris-Sud, Orsay, France; 4 Laboratory for Developmental Studies, Harvard University, Cambridge, Massachusetts, United States of America; 5 AP-HP, Service de Neurologie Pédiatrique, Hôpital Bicêtre, Kremlin Bicêtre, France; 6 Collège de France, Paris, France; University of Oregon, United States of America

## Abstract

All humans, regardless of their culture and education, possess an intuitive understanding of number. Behavioural evidence suggests that numerical competence may be present early on in infancy. Here, we present brain-imaging evidence for distinct cerebral coding of number and object identity in 3-mo-old infants. We compared the visual event-related potentials evoked by unforeseen changes either in the identity of objects forming a set, or in the cardinal of this set. In adults and 4-y-old children, number sense relies on a dorsal system of bilateral intraparietal areas, different from the ventral occipitotemporal system sensitive to object identity. Scalp voltage topographies and cortical source modelling revealed a similar distinction in 3-mo-olds, with changes in object identity activating ventral temporal areas, whereas changes in number involved an additional right parietoprefrontal network. These results underscore the developmental continuity of number sense by pointing to early functional biases in brain organization that may channel subsequent learning to restricted brain areas.

## Author Summary

Behavioural experiments indicate that infants aged 4½ months or older possess an early “number sense” that, for instance, enables them to detect changes in the approximate number of objects in a set. However, the neural bases of this competence are unknown. We recorded the electrical activity evoked by the brain on the surface of the scalp as 3-mo-old infants were watching images of sets of objects. Most images depicted the same objects and contained the same number of objects, but occasionally the number or the identity of the objects changed. As indicated by the voltage potential at the surface of the scalp, the infants' brains reacted when either object identity or number changes were introduced. Using a 3-D model of the infant head, we reconstructed the cortical sources of these responses. Brain areas responding to object or number changes are distinct, and reveal a basic ventral/dorsal organization already in place in the infant brain. As in adults and children, object identity in infants is encoded along a ventral pathway in the temporal lobes, although number activates an additional right parietoprefrontral network. These results underscore the developmental continuity of number sense by pointing to early functional biases in brain organization.

## Abstract 3

Cerebral imaging reveals that human infants are sensitive to numerical quantity at a very early age and that the basic dorsal/ventral functional organization is already in place in the infant brain.

## Introduction

Converging behavioural, brain-imaging, and neurophysiological results suggest that knowledge of number is an evolved competence of the animal and human brain, with a cortical basis in bilateral intraparietal cortex [1–8]. The number sense hypothesis [1] postulates that this cerebral system is available early on during development, possibly during infancy, and guides the learning of numerals and arithmetic in childhood. Indeed, an association of number processing tasks with intraparietal areas has been demonstrated in 4- and 5-y-old children [6,9]. At this age, a change in the cardinality of a set of objects (also called “numerosity”) leads to a response in the right intraparietal cortex, at a location comparable to adults [4,5]. This response cannot be ascribed to a domain-general attentional reaction to novelty inasmuch as it is not seen if the number stays constant while the identity of the objects changes [6].

It is tempting to speculate that this parietal sensitivity arises from a predisposition of parietal cortex for spatial and numerical transformations, possibly present since birth. In subjects below 4 y of age, however, neuroimaging explorations are limited, and the existence of early numerical abilities is mostly based on behavioural results. Habituation and violation-of-expectancy paradigms have revealed a clear sensitivity to large numbers in 4–6-mo-old infants. For instance, infants discriminate sets of eight versus 16 dots, even when nonnumerical parameters such as density and total surface are tightly controlled [10]. In the range of small numbers one, two, and three, however, behavioural evidence remains debated. Initial observations suggested discrimination in infants and even in newborns [11,12], but more recent studies demonstrated that this competence was mostly driven by confounded low-level perceptual dimensions such as continuous amount of stuff [10,13–15]. Nevertheless, Feigenson [16] demonstrated that infants can be driven to attend to numerosity, even in the range from one to three objects, when the sets comprise highly distinctive objects rather than replications of the same object. Moreover, successes at numerical tasks have been observed when the stimuli to be compared are presented in different modalities [17–19], therefore eliminating the possibility of relying on nonnumerical aspects of the stimuli.

Here, we aimed at bringing brain-imaging evidence to bear on the existence of a dedicated number sense system in early infancy. Our experiments probed the infant brain for a specific response to changes in the cardinal of sets of objects, distinct for the response to changes in object identity, and possibly common to small and large numbers. Identifying a brain response to numerosity in infancy would support the hypothesis of a developmental continuity in number sense and significantly extend the current evidence for a cerebral specialization for number, which is currently based almost exclusively on adult and children data. Only a single brain-imaging experiment to date has been conducted on infants' processing of number. Berger et al. [20] used event-related potentials to investigate the cerebral process underlying adults' and 7-mo-olds' reactions to correct and incorrect arithmetic operations (1 + 1 = 2 vs. 1 + 1 = 1). Compared to plausible outcomes, arithmetic violations elicited a cerebral reaction over anterior electrodes both in infants and adults. Because it emerged from the comparison of two arithmetical situations, which differed by their level of plausibility, this reaction probably indexes a general process of violation detection, underlying infants' behavioural response in terms of looking time to such implausible events.

Our experimental design relied on the habituation paradigm, which capitalizes on the phenomenon of neural adaptation: when a stimulus is repeatedly presented, the response of neural populations encoding this stimulus decays progressively over successive trials, but it recovers when a novel item is introduced. In particular, brain areas sensitive to number increase their activation in response to a change in number within a block where most stimuli have the same number [4–6]. Although predominantly used with functional magnetic resonance imaging (fMRI), habituation paradigms have also been successfully applied to young infants using event-related potentials (ERPs) [21,22]. We used ERP adaptation to investigate numerical abilities in 3-mo-old infants. This age is significantly younger than what is seen in most behavioural studies of infant numerical competence, which typically study 4½- to 6-mo-olds [10,13,18,23–26]. Although a few studies have reported number discrimination in newborns [11,12], these studies did not typically incorporate sophisticated controls over all nonnumerical parameters such as size, density, and occupied area.

In addition to probing numerical representation at this early age, we aimed to study whether the infant brain is already anatomically and functionally structured. In adults and 4-y-olds, fMRI adaptation has revealed a double dissociation between ventral regions sensitive to changes in object identity, but not in number, and dorsal regions sensitive to changes in number, but not in object identity [4,6]. Within the visual system, the ventral pathway is thought to be primarily concerned with object identity (“what”), and the dorsal pathway with object location, size, and motor affordance (“where” and “how”) [27]. At 4 mo of age, behavioural evidence suggests that this basic dorsal/ventral organization may be already present, since infants are able to selectively attend either to the identity of objects (e.g., faces) or to their location, yet fail to link these two types of information [28]. In line with these results, a brain-imaging experiment with 6-mo-olds revealed that holding a stimulus in memory relies on different brain mechanisms, depending on the nature of the stimuli, and therefore, presumably, the type of information retained in memory (identity vs. location) [29]. However, the presence of a ventral/dorsal organization in infancy has not been tested directly by looking at the anatomical localization of brain activation.

**Figure 1:**
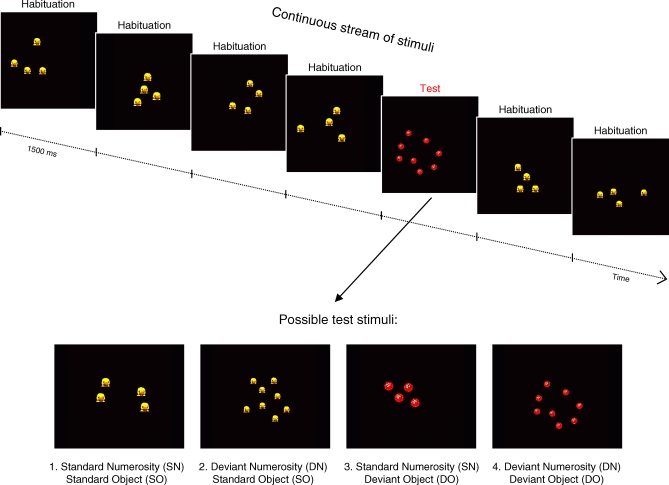
Schematic Description of the Experimental Protocol Infants were presented with a continuous stream of images, each depicting a set of objects. Within a given block, all habituation stimuli shared the same SN and object identity. Occasionally, a test stimulus was inserted that could differ in number and/or object identity. We only report ERPs to those test stimuli, averaged separately for standard versus deviant number trials, and for standard versus deviant object trials.

To address these questions, we recorded event-related potentials from 3-mo-old infants while they were presented with a continuous stream of images, each showing a set of objects. Within a given run, most sets had the same “standard” cardinality and object identity. However, we occasionally inserted test images that could differ from the habituation images in number and/or object identity, thus defining four types of test stimuli (Figure 1).We compared the visual event-related potentials evoked by unforeseen changes either in the identity of objects forming a set, or in the cardinal of this set. Because of the debated possibility of a discontinuity between the small and large number ranges, our tests involved both large and small numbers. Three groups of infants were tested respectively with a pair of small numbers (two versus three), distant large numbers (four versus eight), or very distant large numbers (four versus 12).

## Results

### Dissociated Responses to Number and Object Changes

We examined the event-related potentials evoked by the same critical test images, which were defined as deviant or not, on both the numerical and object-identity dimensions, as a function of their relation to the context provided by the preceding images. ERPs showed a series of waveforms classically observed in infant studies with visual stimuli [21,30]. We observed two successive occipital negativities peaking at 88 and 192 ms after image onset, followed by an ample and long-lasting waveform peaking at 484 ms (P400), which was positive on posterior electrodes and negative on anterior electrodes. In order to evaluate the cerebral responses to changes in number and object identity, we computed the difference between the deviant number and standard number test images, and also between the deviant object and standard object test images. These differences were tested using a cluster-detection algorithm that detects spatiotemporal clusters with a consistent statistical difference over time and space, and assesses their significance against re-randomized data, with a correction for the large search space (electrodes and time points; see Methods). This cluster analysis indicated that the introduction of a change in number or in object identity generated discrimination effects that modulated the duration and amplitude of the P400.

**Figure 2:**
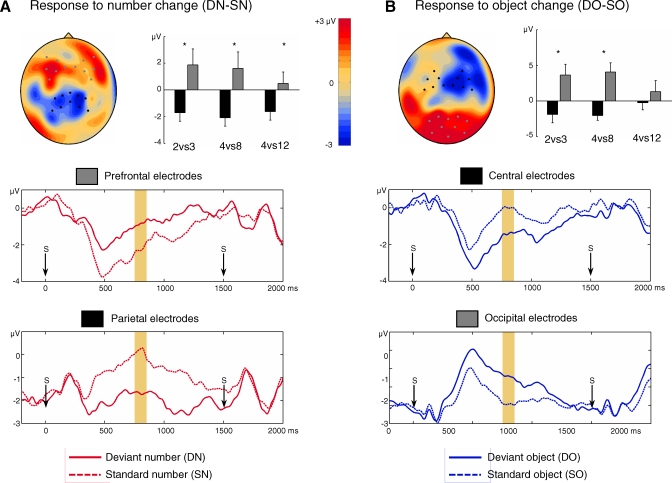
Scalp-Measured Responses to Number Change and to Object Change (A and B) Upper panels show the topography of the difference between the deviant and standard conditions, averaged between 750 to 850 ms after stimulus onset ([A] shows the response to number change; [B] shows the response to object change). Dots mark the location of the electrodes used for ANOVA. Histograms depict effect sizes averaged across these electrodes, for three different groups of infants respectively exposed to numbers two versus three, four versus eight, or four versus 12 (an asterisk [*] indicates *p* < 0.05). Lower graphs show the time course of voltage averaged over the groups of electrodes used in the ANOVAs. Arrows indicate stimulus onsets (every 1,500 ms).

For number change, a significant effect (corrected *p* = 0.044; uncorrected *p* = 0.0073) was due to more-negative voltages on number change compared to same-number trials over a cluster of 13 bilateral parietocentral electrodes (712–1,196 ms after stimulus onset), and more-positive voltages over two left frontal electrodes (992–1,196 ms). To further explore the number-change effect, voltages were averaged on a 100-ms time window (750–850 ms) and four groups of electrodes corresponding to the negative and positive maxima of this effect over parietal and prefrontal areas, and the symmetrical groups on the other hemisphere (see Methods and Figure 2A for the localisation of the electrode groups). These values were entered in two four-factors ANOVAs (number size, left vs. right hemisphere, parietal vs. prefrontal electrode group, and number change or object change as the last factor). On these electrodes, number change interacted significantly with the electrode group (*F*(1,33) = 18.7, *p* = 0.0001), but object change did not (*F* < 1). The effect did not differ significantly across hemispheres (interaction: *F* < 1) and was significant over both the left and the right electrode groups (left hemisphere: *F*(1,33) = 21.1, *p* < 0.0001; right hemisphere: *F*(1,33) = 8.3, *p* = 0.0069). The number-change effect was also consistent across groups as it did not interact with number size (*F*(2,33) < 1) and was significant within each experimental group, including the small-number group (four vs. 12: *F*(1,11) = 6.6, *p* = 0.026; four vs. eight: *F*(1,11) = 5.7, *p* = 0.036; and two vs. three: *F*(1,11) = 7.9, *p* = 0.017).

Object change also yielded a significant effect (corrected *p* = 0.035; uncorrected *p* = 0.0054), but slightly faster and with a distinct topography including a right anterior negative cluster (13 electrodes, 672–972 ms) and a bilateral temporooccipital positive cluster (ten electrodes, 636–868 ms). Similar to the analysis of the number effect, an ANOVA was performed on the voltage averaged during the same 100-ms time window and on four groups of electrodes (two groups located at the maxima of the dipole of the object-change response on occipital and central areas, and their symmetrical counterparts; see Figure 2B for the localisation of the electrode groups). On these electrodes, object change interacted with electrode localization (*F*(1,33) = 13.1, *p* = 0.001), whereas number change did not (*F*(1,33) = 1.2, *p* = 0.28). Voltages were more negative for deviant objects on central electrodes, and more positive on occipitotemporal electrodes. This effect did not interact with hemisphere (*F*(1,33) = 1.4 *p* = 0.24), and it reached significance over both the left and the right electrode groups (left hemisphere: *F*(1,33) = 7.0, *p* = 0.012; right hemisphere: *F*(1,33) = 16.9, *p* = 0.00024). The object change effect also did not interact with number size (*F*(2,33) = 1.4, *p* = 0.26). However, further analyses revealed a significant response to object change only in the two groups that saw the smallest numbers (two versus three group: *F*(1,11) = 6.7, *p* = 0.025; four versus eight group: *F*(1,11) = 13.4, *p* = 0.004); but not in the four versus 12 group (*F* < 1). A likely interpretation is that objects were presented at a smaller physical size in the latter condition and thus were harder to discriminate (see [4] for a similar observation).

Together, these results establish a double dissociation between the processing of number and of object identity at the scalp level: some electrode groups show an effect of number change, but not of object change, whereas others show the converse response. The dissociation indicates that neither of these responses can be ascribed solely to domain-general mechanisms such as attention to novel events.

### Cortical Sources

**Figure 3:**
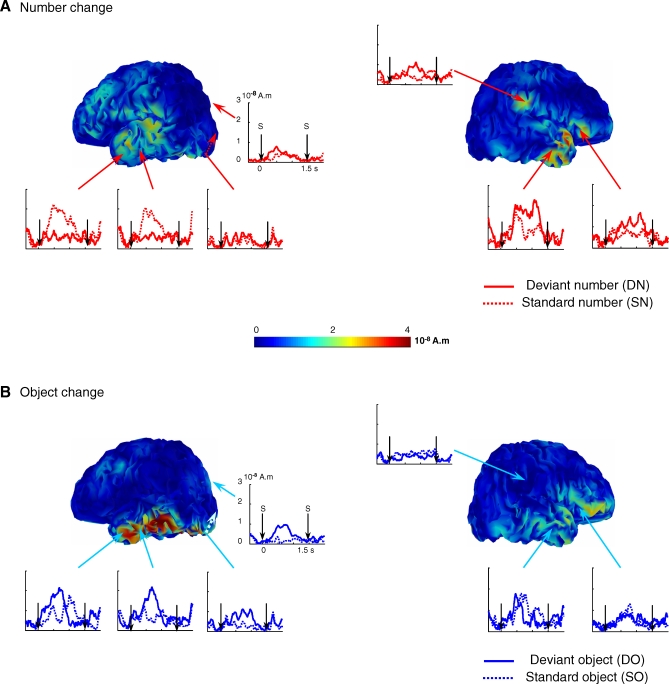
Cortical Source Reconstructions (A and B) The sources of the deviant–standard differences are presented on a 3-mo-old 3-D brain at 832 ms after stimulus onset. Time courses were extracted from different brain areas for standard and deviant conditions. In the deviant object condition (B), areas of the left temporal lobe were more activated than in the standard object condition. In the deviant number condition (A), a right parietoprefrontal network was activated, in addition to a right anterior temporal response. Number change also led to a deactivation in the left temporal areas responding to object deviance, perhaps because the introduction of a number deviant inhibited the mechanisms encoding object deviance.

Although event-related potentials have low spatial resolution, they can provide coarse information about brain localization, particularly given the dense sampling of the infant head provided by our 65-electrode net. We took advantage of the anatomical images obtained from our previous magnetic resonance experiments with infants [31,32] to compute a detailed model of the infant head and cortical folds (see Figure 3). We then used this forward model to reconstruct a plausible distribution of the cortical origins of our scalp recordings, using distributed cortical source modelling and a minimum norm constraint (see Methods). The reconstructed activations, while probably accurate only to within 1 or 2 cm, indicated a double dissociation of number and object identity at the cortical level (see Figure 3). Object change activated a stream of left ventral temporal regions, starting in posterior occipitotemporal regions around 300–400 ms and with a durable activation up to 800–1,000 ms in anterior temporal as well as posterior occipitotemporal regions. Conversely, number change led to the activation of a right network pertaining to the dorsal pathway, including the right inferior parietal and right inferior frontal region. We also observed effects of number change in temporal regions. An antagonistic relation was observed whereby number change led to a decrease in the left anterior temporal regions previously associated with object change, and a concomitant increase in right anterior temporal activation (see Figure 3).

### Comparison across Small and Large Number Ranges

We found a response common to small and large numbers on the scalp; we also searched the data for possible differences between them. We first examined whether the timing of the number-change effect varied with the range of numbers tested. An ANOVA was run on a larger time window (650–1,250 ms) spanning the whole duration of the effect as identified by the cluster analysis, on the groups of electrodes defined by the number-related analysis, including the same factors as previously analyzed (number size, hemisphere, electrode group, and number change) and a supplementary factor of time (six successive 100-ms–long time windows). The effect (interaction between electrode group and number change) did not interact with time or with number size (all *F*s < 1), even when the two groups of infants that were presented with large numerosities were grouped together (*F*s < 1). A cluster analysis directly comparing the responses to number change in the small-number range (two versus three group) versus the large-number range (four versus eight and four versus 12 groups) also did not identify any effect (corrected *p* > 0.42, uncorrected *p* > 0.11). Thus, our data do not show any discontinuity between small and large numbers.

## Discussion

By looking at brain electrical activity in 3-mo-old infants, our results establish a double dissociation between number and object identity processing. Visual evoked potentials were recorded as infants were presented with images of sets of objects, most of them depicting the same number of the same objects, but with occasional deviants in object identity and/or number. At the scalp level, some electrode groups showed an effect of number change, but not of object change, whereas others showed the converse response. Those brain responses reveal that infants are sensitive to both object identity and number by age 3 mo. For object identity, our results merely provide additional support to a considerable amount of previous behavioural evidence for visual object processing in infancy, including in newborns [33–35]. For number, however, our experiment goes beyond previous behavioural research by demonstrating numerical discrimination at an age younger than most previous reports [10,13,18,25,26], and by including strict controls over nonnumerical parameters that were typically lacking in previous experiments at this age [12,24].

The observed dissociation between object identity and number is particularly crucial for the interpretation of the functional role of the observed brain responses. It indicates that neither of them can be ascribed solely to domain-general mechanisms such as attention to novel events. Detection of rare events has been associated with a series of late waveforms in infants, and particularly a large negative deflection culminating 400–800 ms after stimulus onset on the anterior electrodes (Nc) [36–38]. Similarly, Berger et al. [20] observed a negative anterior waveform when 7-mo-old infants watched a movie that included an arithmetic violation (1 + 1 = 1 or 2 − 1 = 2). Although direct comparison of these results with ours is made difficult by the occasional use of different references for voltages, the domain-general Nc response bears some resemblance with the response we observed after changes of objects, which includes a negative component on frontal electrodes. The response to changes in number, however, is clearly distinct from this domain-general Nc. Our source analysis further suggests that neither of these components originates solely from an anterior attention network, which is considered to be the source of the Nc component [37], but that both our responses involve distributed and distinctly located sources.

The observed dissociation between object- and number-based responses also allows us to exclude simple interpretations of our results as artefacts of the experimental procedure. In particular, although parents were allowed to see the stimuli, it is very unlikely that the reaction we observed was a reaction to a parental signal rather than a reaction to our stimuli. First, the onset of the brain reaction occurred before 500 ms, which leaves little time for a whole chain of parental reaction, parent-to-infant signalling, and infant brain activation to occur. Second, most crucially, given the dissociation in ERPs associated with number and object change, it seems very improbable that all parents would have differentiated the two types of changes in a consistent way so as to elicit, through an unknown, yet differentiated feedback, a consistently distinct brain response in all infants.

### Dorsal and Ventral Pathways in Infants

Although they should be interpreted cautiously, as they represent only a tentative model with coarse spatial accuracy, our source reconstructions suggest, at a minimum, that distinct cortical pathways already exist in 3-mo-old infants for processing number and object identity. The sources of the object-change effect were located along the left temporal cortex, with an antagonistic response in the right temporal cortex. These results mesh well with fMRI observations from children and adults, where object-change responses have been recorded in the inferotemporal cortex, particularly in the left hemisphere [4,6]. In the present experiment, objects were defined both by their shape and by their colour, and therefore the observed response to object change could involve lower-level colour- and shape-sensitive areas as well as object-sensitive areas. Indeed, examination of the source reconstruction results brings support to this interpretation, since an entire stream of ventral occipitotemporal areas was activated in cascade in response to the presentation of deviant objects. The posterior areas, which responded first, might have encoded low-level features of the stimuli such as shape or colour, whereas the later activation of more anterior areas might relate to an encoding of object identity. Note, however, that the surface ERP component responding to object change was probably not sensitive solely to changes in colour, since it did not appear in the large-number condition in which the objects were smaller and therefore hardly discriminable in shape, but still markedly different in colour.

In contrast to changes in object identity, changes in number activated a parietoprefrontal network in the right hemisphere. Although bilateral areas have been associated with number processing in adults, the present lateralization to the right hemisphere is consistent with previous studies that suggest a greater right lateralization of intraparietal responses to number in 4-y-olds than in adults [6]. The activation of the left inferior parietal region increases with age in older children [39,40], and our results suggest that it might not be dominant in the first year of life.

In addition to activating a right parietoprefrontal network, changes in number elicited a decreased response within the left temporal region that was responsive to object change (as well an increase in the opposite right temporal region). This aspect of our results suggests that there may be an antagonistic relation between the dorsal network for number and space and the ventral network for object identity. Although unexpected, this tentative conclusion meshes well with several previous behavioural studies that observed a drop in infants' performance when object identity and either numerical or spatial information have to be jointly processed and integrated. For instance, Xu [41] and Xu and Carey [42] observed that infants were unable to use object-identity information to infer numerosity: when two distinct objects emerged successively from behind an occluder, infants were clearly able to discriminate these two objects, but they were not surprised if the occluder later dropped to reveal just one object. Furthermore, Mareschal and Johnson [28] explicitly demonstrated a competition between memory for object features (“‘what”‘) versus spatiotemporal trajectory (“‘where”‘) depending on the category of visual stimuli used. When the stimuli were faces, infants detected changes in their identity, but not their position, whereas when they were manipulable toys, infants detected changes in location, but not in identity [28]. Furthermore, Southgate et al. [29] observed a dissociation in the brain responses of 6-mo-old infants as they were holding either a toy or a face in memory. These results, together with our source localizations, support the hypothesis that during infancy, information pertaining to the “‘what”‘ and “‘where”‘ pathways is already processed by distinct networks that are initially not fully integrated in a coherent behaviour, but rather may interact according to an antagonistic mode. Further research will be needed to confirm this antagonistic effect and probe the roles of attention, language, and prefrontal cortex development in overcoming it. Some researchers have suggested that a full integration may not occur until much later during childhood [43].

### Developmental Continuity of Number Representations in the Dorsal Pathway

Our results, which are based on a measure of infant cerebral activity, differ in part from previous conclusions based on behavioural results. We observed a shared brain response to numerical changes in both small (two vs. three) and large (four vs. eight and four vs. 12) number ranges. This finding is in apparent conflict with previous behavioural results suggesting that even 6-mo-old infants are unable to discriminate small numbers two and three when nonnumerical parameters are appropriately controlled [13,44,45], and are unable to discriminate ratios of 2:3, even in the large-number range, until 9 mo of age [46].

At this age, infants are thought to possess two separate systems conveying numerical information in the small- (<4) and large-number range, respectively. In the large-number range, numbers are represented by analogical internal magnitudes. In the small-number range, infants track sets of one to three items by means of attentional indexes attached to each object, and they can use one-to-one correspondence on these indexes to detect the absence or sudden apparition of objects [13,47]. With respect to this theoretical background, our results raise three questions: (1) why did we observe a capacity of the dorsal system to respond to both small and large numbers? (2) why did we not observe a distinct brain response to small numbers? and (3) why do brain measures seem to be more sensitive than behavioural measures?

### A Shared System Dealing with Both Small and Large Numbers

Although behavioural results demonstrate the existence of a specific system for small numbers, no published study contradicts our present findings that the system of analog magnitude representation, which underlies infants' numerical competence for large numbers, can also respond to small numbers. In fact, many adult and animal studies reveal a continuity in behaviour between small and large numbers, suggesting that the analog system extends to small numbers. For instance, Cordes et al [48] found the same Weber signature across small and large numbers in a task adapted from the animal literature, in which human adults were required to tap a given number of times without counting. Contrary to the prediction of the small number system, responses showed some variability in the small-number range, in continuity with the large-number range. In tapping tasks, animals show a similar behaviour, with no discontinuity between small and large numbers [49]. In infants, a set-size signature characteristic of the object tracking system is obtained mostly in one type of task in which items are shown successively or retrieved successively from a box [50,51]. Hauser et al. [52] observed a similar set-size limit in untrained rhesus monkeys tested with a single trial in semiwilderness, but Beran [53] found no such discontinuity when using a similar task with multiple trials in trained laboratory animals: monkeys could select the larger of two sets based on their number, with a ratio-dependent performance and no discontinuity between small and large numbers. Under conditions of simultaneous presentation of a set of objects, as tested in the present work, monkeys' analog representation always appear to extend to small numbers. For instance, Brannon and Terrace [54,55] observed ratio-based generalization from small to large numbers: after having been trained on the comparison of small quantities (one to four), rhesus monkeys were able to generalize this training to larger numbers (up to nine).

At the brain level, neurons sensitive to numerosity have been found in the intraparietal sulcus as well as in the prefrontal lobe of monkeys. These neurons encode quantities from one to 30 objects and show a seamless increase in their tuning width with numerosity, corresponding to Weber's law, without any sign of a discontinuity at the boundary between numbers smaller or larger than three [7,56]. Similar observations have recently been made in another population of number-sensitive neurons located in lateral intraparietal area [57]. Our results accord with these observations, and suggest that human infants share with nonhuman primates an analog representation of numerosities that extends seamlessly across small and large numbers alike.

### Absence of Brain Correlates of the Small-Number System

Given the behavioural evidence for a distinct small-number system in infancy, one might wonder why we did not observe an additional cerebral response in the small-number range. Several aspects of our design might have precluded this possibility, however. First, behavioural results show that infants can discriminate small numbers via an object tracking system, but that this competence disappears when the objects are identical and nonnumerical aspects of the sets, such as object size and density, are controlled for [58]. These factors were tightly controlled for in the present experiment, and thus our design may have effectively cancelled any response of the small-number system. Second, even if this system had been reactive, the neural habituation method that we used may not be appropriate to detect it. Repetition suppression occurs in neural populations tuned to the stimulus value presented repeatedly during the habituation phase. Thus, the habituation method is most appropriate to detect neural populations that encode an explicit representation of the dimension being tested. The object tracking system, however, is thought to represent numerical information only in an implicit way: in this system, there is no summary representation of “two”; instead, infants form a mental model of two objects by recruiting two attentional indexes or “object files” [47,59,60]. This system, therefore, may not show a repetition suppression effect and may remain invisible to the neural habituation method. In brief, our experiment was not targeted at detecting the cerebral bases of an object tracking mechanism. Consequently, the absence of a specific effect for small numbers in our results does not exclude the existence of such system, which may perhaps be detected by other means.

### Contrasting Cerebral and Behavioural Measures

Finally, our event-related potentials revealed a capacity for discriminating numerosity, whereas behavioural studies typically show infants failing in similar conditions. Hence, although we observed a positive reaction to changes in number in brain activity, 5-mo-old infants are not able to discriminate numerosities when images were presented at the rate of one every 1,500 ms as here, but needed a longer presentation of 2,000 ms to succeed in a behavioural experiment [26]. Furthermore, we observed a reaction for numbers separated by a ratio of 2:1 (four vs. eight) and even 3:2 (three vs. two), although positive discrimination of 3:2 ratios is not achieved until 9 mo of age in behavioural measures [10,46]. Whereas behaviour is often a composite measure reflecting the combined effects of several processing stages, brain measures can provide a purer index of a given level of representation [61] and can track the response of a given system even when it does not lead to an overt behavioural response. Many examples of ERP-behaviour dissociations exist in the adult literature. For instance, high-density ERPs demonstrate a series of processing stages of subliminal visual stimuli that subjects deny seeing [62]. In a training task where subjects learn to attend to subtle phonetic differences, ERP evidence for stimulus discrimination (mismatch negativity) may appear as much as 24 h before a change in overt behaviour occurs [63]. These experiments exemplify the fact that cerebral measures can be more sensitive than behavioural measures.

A second factor, more specific perhaps to the developmental context, is that when a conflict exists between several levels of representations, infants and young children may lack the ability to resolve the conflict and integrate all of their sources of knowledge into a coherent behaviour. As a result, behavioural measures may not fully reflect the infant's competence. For instance, in the classical object permanence task, infants appear to lack knowledge of hidden objects when tested with reaching measures, but not when tested with looking time or eye-orienting methods [34,35,64,65]. In the case of small numbers, object-tracking representations may be so salient that they determine behaviour even when the analog quantity system is in possession of more-advanced information concerning numerical quantity. As discussed above, object identity and set numerosity pertaining to the ventral and the dorsal pathway may be not fully integrated during infancy. Furthermore, ERP responses to object and number change occur relatively late, with a peak around 800 ms, much later than the discrimination responses observed at the same age with auditory phonetic stimuli (around 200–400 ms for phonetic mismatch [22]) or visual faces (around 300 to 700 ms [38,66]). This slowness, perhaps related to the small size of the objects presented, may explain that numerical competence is not always expressed in behavioural measures such as looking time, which may be driven by faster computations of more salient perceptual dimensions. In particular, stimuli need to remain present for a longer time in order for infants to react to number [26]. Whereas ERP measures detect the on-line brain response to numerosity, more time may be needed for this response to guide overt behaviour.

We should also underscore the fact that although the stimuli used in behavioural and ERPs experiments can be similar, the constraints related to each type of procedure lead to important differences in experimental settings. Whereas behavioural results are based on a few measures obtained after several minutes of habituation to one type of stimulus, we recorded ERP to tens of object and number changes embedded in a continuous flow of standard stimuli. Such fast recurrent presentation provides more evidence to the infant about the value of the standard numerosity, and repeated measurements may also confer a greater sensitivity to ERP experiments. Both factors may ultimately explain why we observed a reaction to numbers separated by a smaller ratio (2:3), and presented at a faster pace.

### Conclusion

Altogether, our results reveal a system representing both small and large numbers in infancy. They suggest developmental continuity, with a right parietal involvement for number and a basic ventral/dorsal organization already present at 3 mo of age. The parietal number system may constitute the neural substrate of infants' initial numerical competence, which increases in precision in the course of development and possibly guides the acquisition of arithmetic and more elaborate mathematical concepts.

## Methods

### Stimuli.

High-density ERPs were recorded while infants were presented with a continuous stream of images, each depicting a set of colourful animal-like objects on a black background. Stimuli were projected onto a screen measuring 100 × 80 cm, and the infant was seated on a caretaker's lap at an approximate distance of 80 cm from the screen. Two categories of stimuli were used, respectively labelled “habituation” and “test.” Within a block, all habituation stimuli had the same number and depicted the same object. Test stimuli occasionally differed from the habituation stimuli in their number and/or in the identity of the object used, thus defining four types of test stimuli (see Figure 1).

### Control over nonnumerical parameters.

In habituation experiments, it is important to control for nonnumerical parameters, such as the position of the objects and the physical parameters of the image, in order to ensure that a positive result can only be attributed to the discrimination of numbers rather than to the variation of any nonnumerical parameter. We applied a strategy similar to previous publications [4–6,10,67].

The stimuli were automatically generated using a variant of our laboratory's numerosity stimulus generation programs, which are described in depth at http://rd.plos.org/pbio.0060027 (78 KB DOC). The position of the objects and the physical parameters of the image (intensive parameters: object surface size, average area devoted to each object; extensive parameters: total luminance, total occupied area) varied across stimuli, following different rules for the habituation and test stimuli. For the test stimuli, on the one hand, the extensive parameters of the display (total luminance and total occupied area) were kept constant on average. Therefore, if infants encoded some extensive parameter of the stimuli rather than the cardinality of the sets, they would have the same reaction to all test stimuli. For the habituation stimuli, on the other hand, intensive parameters were kept constant on average. They were completely uncorrelated to number, as they were randomly drawn from a uniform distribution, ideally ranging between the minimal and maximal value these parameters take for the test stimuli (see Figure S1). If infants only encoded some intensive parameter of the stimuli, their reaction to the test stimuli should be independent of the numerical habituation context.

In the analysis of the results, we considered the difference between deviant and standard test stimuli in various numerical contexts, where two numerosities took alternatively the roles of deviant and standard numerosity. Reactions to intensive parameters would generate effects depending only on the numerosity of the test stimulus presented, not on the numerical habituation context (no effect of the deviant/standard factor). On the other hand, reactions to extensive parameters would be the same over all test stimuli, independent of their numerosity. Therefore, reactions to the number-change factor can only be imputed to numerosity, and not to the low-level attributes of the stimuli.

### Procedure.

The total experiment consisted of eight blocks of 16 trials each, in which each trial consisted in the initial presentation of a variable number of habituation stimuli (two to five images) followed by a single test stimulus. Within each block, images were presented continuously, at the rate of one image every 1,500 ms, and no cue indicated the presence of a test stimulus or the beginning of a new trial. When the infant looked away from the screen, the experiment was stopped, the infant's gaze was attracted to the screen, and then additional habituation stimuli were presented before a test stimulus appeared.

The experiment ended after eight blocks, or when the infant showed signs of fussiness.

Throughout the whole experiment, each infant was presented with two different numbers whose respective roles (either deviant or standard) switched each time a new block started. Three different pairs of numbers were used in three different groups of infants: very distant large numbers (four versus 12), distant large numbers (four versus eight), and small numbers (two versus three). In order to maximize the infants' attention, in each of the eight blocks a new standard object was selected. The deviant object was chosen so that its shape and colour would be maximally different from the standard object.

### Participants.

Thirty-six healthy infants were included (14 females; mean age 103 d, range 92 to 124 d). Additional infants were rejected because of fussiness (77), excessive movements (31), excessive sweating artefacts (6), electrode net degradation (16), or other technical failure (3). The study was approved by the regional ethical committee for biomedical research, and parents gave their written informed consent.

### ERP recording and signal processing.

Electroencephalography (EEG) was digitized continuously at 250 Hz using a 65-electrode geodesic electrode net (EGI) referred to the vertex. The recording was first digitally filtered between 0.5 and 20 Hz. For each trial, we then extracted an epoch starting 400 ms before the presentation of the test stimulus and lasting 2,000 ms after the onset of the test stimulus. Electrodes contaminated by eye or motion artefacts were automatically rejected, and trials with more than 25 contaminated electrodes were rejected. The remaining trials (on average 61.6 per participant; range 30 to 148) were averaged to obtain ERPs in each of four trial types (DN: deviant number; SN: standard number; DO: deviant object; SO: standard object). Note that experimental design crossed the two variables of number change and object change, so that in principle, we could have computed ERPs within smaller subcategories of trials (e.g., DN with or without a concomitant change in object identity). In practice, however, too few trials were available, so that only the main effects of object change and number change could be studied. ERP averages were digitally transformed to an average reference, corrected for eventual slow artefacts by removing a linear trend on the whole segment, baseline corrected on the 200 ms preceding the onset of the test stimulus, and finally spatially smoothed by convolution with a Gaussian, with a standard deviation corresponding to the distance between electrodes on an infant's head, e.g., about 1.5 cm.

### Statistical analysis: Randomization procedure and ANOVAs.

In order to evaluate the cerebral responses to changes in number and object identity, we computed the difference between the DN and SN test images, and also between the DO and SO test images. Computing these differences cancelled out all the brain activity resulting from processes that are common between the two conditions, such as low-level perceptual processes, or processes resulting from the presentation of the previous habituation stimuli.

A customized cluster analysis coupled with a randomization procedure was used to identify effects and assess their level of significance after correction for the large number of electrodes and time points tested. The two conditions of interests were first compared separately, using Wilcoxon signed-rank test, for each electrode and each time sample. Levels of probability obtained were normalized into *Z*-scores, and then thresholded at ±1.96 (*p* = 0.05 two-tailed). These thresholded values were used to define spatiotemporal clusters of activation present for an extended time period and on contiguous electrodes. The entire time period extending between the onset of the test stimulus and the onset of the next habituation stimulus (1,500 ms) was used to detect the clusters. First, our procedure pooled above-threshold samples corresponding to spatially contiguous electrodes or adjacent time samples, separately for positive and negative above-threshold *Z*-scores, thus defining several positive and negative clusters. Each cluster was then attributed a weight equal to the sum of the absolute value of the *Z*-scores of all of its constitutive samples. Because our data were transformed to an average reference, significant effects were expected to present two near-simultaneous ERP components of opposite signs over two distinct electrode sets. Therefore, for each time sample, the total effect strength was measured as the sum of the weights of the largest positive and negative clusters that were found at this time.

To evaluate the significance of the effects obtained, we then recomputed the same analysis on 5,000 sets of randomly permuted data, for which no significant effect is expected. A permutation was defined by randomly attributing the label “deviant” or “standard” to the two conditions of interest for each subject. For each permutation, we extracted the distribution of effect strength (number of pairs of clusters of each strength), as well as the value of the maximum effect strength for the total time interval. Uncorrected *p*-values were obtained by averaging the distribution of effect strength over all the permutations, and then taking the rank of the real experimental data within this average distribution. Similarly, corrected *p*-values were given by the rank of the experimental data within the distribution of the maximal effect strength, divided by the total number of permutations [68].

The previous method allowed selecting the time windows and clusters of electrodes that were significantly affected by our experimental conditions. Possible effects of group and hemisphere on these effects were further explored with analyses of variance (ANOVAs) in order to assess (1) the consistency of the effect over different groups, (2) the independence of responses to number changes and object changes, and (3) possible asymmetries in the cerebral response. Voltages were extracted for each of the four experimental conditions (DN, SN, DO, and SO) within symmetrical groups of electrodes and a fixed time window (750–850 ms), identified by the above cluster analysis as a common window for number and object change effects. The electrode groups chosen corresponded to the six electrodes located at the negative and positive maxima of the effect, as well as their symmetrical electrodes (identical to themselves for medial electrodes). This way, two symmetrical groups of ten parietal electrodes and 12 prefrontal electrodes were defined for the number-change effects; and two groups of ten occipital electrodes and 12 central electrodes were defined for the object-change effect. Average voltages were then entered into two distinct ANOVAs testing respectively for numerical change and object change, with one between-subject factor (number size: 2/3, 4/8, and 4/12) and three within-subject factors of hemisphere, electrode group localization, and deviance (change or no change). Finally, in order to evaluate the possibility that these number pairs caused deviancy effects at different moments in time, another ANOVA, with a supplementary factor of time, tested six successive 100-ms–long time windows (from 650 to 1,250 ms).

### Source modelling.

Cortical current density mapping was obtained using a distributed model consisting of 10,000 current dipoles. Dipole locations and orientations were constrained to the cortical mantle of a generic head and brain model built from a 3-mo-old infant MRI anatomy using the BrainVisa software package (http://brainvisa.info/). The geometry of the EEG sensor net was then warped to the head mesh. EEG forward modelling was computed using an overlapping-sphere analytical model in which all sphere and conductivity parameters were adjusted to the typical tissue properties of the infant head [69]. Cortical current maps were computed from the EEG time series using a linear inverse estimator (weighted minimum-norm current estimate). All these procedures were conducted with the BrainStorm Matlab toolkit (http://neuroimage.usc.edu/brainstorm).

## Supporting Information

Alternative Language Abstract S1 French Translation of Abstract by VI (21 KB DOC)

Figure S1 Distribution of the Nonnumerical Parameters of the Stimuli Schema illustrating how the physical parameters of the stimuli were controlled. Values corresponding to the pair of large distant numbers (four vs. 12) are given as an example. Left, control over the luminance of individual items (intensive parameter) versus of the whole display (extensive parameter). The total luminance stayed constant for all the test stimuli, independent of their number, whereas the intensive parameter (surface size of the objects) was constant on average for habituation stimuli. Thus, the average amount of change in the size of the objects was exactly the same on standard trials, where the numerosity at test equals the numerosity during habituation, than on deviant trials, where they differ. If we observe a significant cerebral response to number changes (difference between deviant number and standard number), this result can only be explained by number change and not by object size or total luminance. Right, similar control over total occupied area (extensive parameter) versus mean area occupied by each object (intensive parameter). In this case, a supplementary variability was introduced in the images, as the positions of the objects were chosen randomly by a program to make the arrays look irregular. Despite this variability, controls ensured that (1) test stimuli were minimally variable across numerosities in terms of total occupied area; (2) the test stimuli were not systematically more similar to the habituation stimuli of the same numerosity, as compared to stimuli of a different numerosity.
(88 KB DOC)

## Abbreviations

EEG: electroencephalogram
ERP: event-related potential
Nc: negative component 400–800 ms after stimulus onset
