# Supplementary material for: Distinct Cerebral Pathways for Object Identity and Number in Human Infants
Source: PLoS Biol. 2008 Feb 5;6(2):e11. doi: 10.1371/journal.pbio.0060011 (PMC2225438; doi:10.1371/journal.pbio.0060011)
Supplement: Supplementary file 1 [file pbio.0060011.sd001.doc]

Traduction de l’abstract en français

Quelle que soit leur culture et leur niveau d’éducation, tous les humains possèdent des compétences numériques intuitives. Les études effectuées jusqu’ici reposent sur des méthodes comportementales et suggèrent que ces compétences sont présentes dès les premiers mois. Ici, nous utilisons une méthode d’imagerie cérébrale pour montrer que le cerveau des bébés encode les informations numériques ainsi que les propriétés des objets séparément, dès l’âge de 3 mois. Nous comparons les potentiels visuels évoqués par des changements imprévus introduits sur des images représentant des ensembles d’objets, au niveau de l’identité des objets ou du nombre d’objets présents. Chez l’adulte, et l’enfant de 4 ans, le sens des nombres repose sur un système bilatéral d’aires du sillon intrapariétal, différent du système ventral occipito-temporal codant pour l’identité des objets. La topographie des champs de voltage sur le scalp et les modèles de sources cérébrales reconstruites révèlent une même dissociation chez les bébés de trois mois : les changements d’objets activent des aires temporales ventrales, tandis que les changements de nombres impliquent en plus un réseau parieto-prefrontal. Ces résultats soulignent le fait que le sens des nombres se développe de manière continue, en mettant en évidence un biais précoce dans l’organisation du cerveau, qui pourra ensuite diriger les nouvelles compétences acquises vers un ensemble restreint d’aires cérébrales.
