# Supplementary material for: Distinct Cerebral Pathways for Object Identity and Number in Human Infants
Source: PLoS Biol. 2008 Feb 5;6(2):e11. doi: 10.1371/journal.pbio.0060011 (PMC2225438; doi:10.1371/journal.pbio.0060011)
Supplement: Supplementary file 2 [file pbio.0060011.sg001.doc]

## Supplementary Figure

**figure S1 legend.** Schema illustrating how the physical parameters of the stimuli were controlled. Values corresponding to the pair of large distant numbers (4 vs 12) are given as example. Left, control over the luminance of individual items (intensive parameter) versus of the whole display (extensive parameter). The total luminance stayed constant for all the test stimuli, independently of their number, while the intensive parameter (surface size of the objects) was constant on average for habituation stimuli. Thus, the average amount of change in the size of the objects was exactly the same on standard trials, where the numerosity at test equals the numerosity during habituation, than on deviant trials where they differ. If we observe a significant cerebral response to number changes (difference between deviant number and standard number), this result can only be explained by number change and not by object size or total luminance. Right, similar control over total occupied area (extensive parameter) versus mean area occupied by each object (intensive parameter). In this case, a supplementary variability was introduced in the images, as the positions of the objects were chosen randomly by a program to make the arrays look irregular. Despite this variability, controls ensured that (1) test stimuli were minimally variable across numerosities in terms of total occupied area; (2) the test stimuli were not systematically more similar to the habituation stimuli of the same numerosity, as compared to stimuli of a different numerosity.
